# Biochemical Characterization of UDP-*N*-acetylmuramoyl-L-alanyl-D-glutamate: *meso*-2,6-diaminopimelate ligase (MurE) from *Verrucomicrobium spinosum* DSM 4136^T^

**DOI:** 10.1371/journal.pone.0066458

**Published:** 2013-06-13

**Authors:** Sean E. McGroty, Dhivya T. Pattaniyil, Delphine Patin, Didier Blanot, Arvind C. Ravichandran, Hironori Suzuki, Renwick C. J. Dobson, Michael A. Savka, André O. Hudson

**Affiliations:** 1 The Thomas H. Gosnell School of Life Sciences, Rochester Institute of Technology, Rochester, New York, United States of America; 2 The School of Chemistry and Materials Science, Rochester Institute of Technology, Rochester, New York, United States of America; 3 Univ Paris-Sud, Laboratoire des Enveloppes Bactériennes et Antibiotiques, Institut de Biochimie et Biophysique Moléculaire et Cellulaire, Orsay, France; 4 Centre National de la Recherche Scientifique, Orsay, France; 5 Biomolecular Interaction Centre, School of Biological Sciences, University of Canterbury, Christchurch, New Zealand; 6 Department of Biochemistry and Molecular Biology, Bio21 Molecular and Biotechnology Institute, The University of Melbourne, Parkville, Victoria, Australia; Institut Pasteur Paris, France

## Abstract

*Verrucomicrobium spinosum* is a Gram-negative bacterium that is related to bacteria from the genus *Chlamydia*. The bacterium is pathogenic towards *Drosophila melanogaster* and *Caenorhabditis elegans*, using a type III secretion system to facilitate pathogenicity. *V. spinosum* employs the recently discovered l,l-diaminopimelate aminotransferase biosynthetic pathway to generate the bacterial cell wall and protein precursors diaminopimelate and lysine. A survey of the *V. spinosum* genome provides evidence that the bacterium should be able to synthesize peptidoglycan *de novo*, since all of the necessary genes are present. The enzyme UDP-*N*-acetylmuramoyl-l-alanyl-d-glutamate: *meso*-2,6-diaminopimelate ligase (MurE) (E.C. 6.3.2.15) catalyzes a reaction in the cytoplasmic step of peptidoglycan biosynthesis by adding the third amino acid residue to the peptide stem. The *murE* ortholog from *V. spinosum* (*murE*_Vs_) was cloned and was shown to possess UDP-MurNAc-l-Ala-d-Glu:*meso*-2,6-diaminopimelate ligase activity *in vivo* using functional complementation. *In vitro* analysis using the purified recombinant enzyme demonstrated that MurE_Vs_ has a pH optimum of 9.6 and a magnesium optimum of 30 mM. *meso*-Diaminopimelate was the preferred substrate with a *K*_m_ of 17 µM, when compared to other substrates that are structurally related. Sequence alignment and structural analysis using homology modeling suggest that key residues that make up the active site of the enzyme are conserved in MurE_Vs_. Our kinetic analysis and structural model of MurE_Vs_ is consistent with other MurE enzymes from Gram-negative bacteria that have been characterized. To verify that *V. spinosum* incorporates diaminopimelate into its cell wall, we purified peptidoglycan from a *V. spinosum* culture; analysis revealed the presence of diaminopimelate, consistent with that of a bona fide peptidoglycan from Gram-negative bacteria.

## Introduction

**Figure 1:**
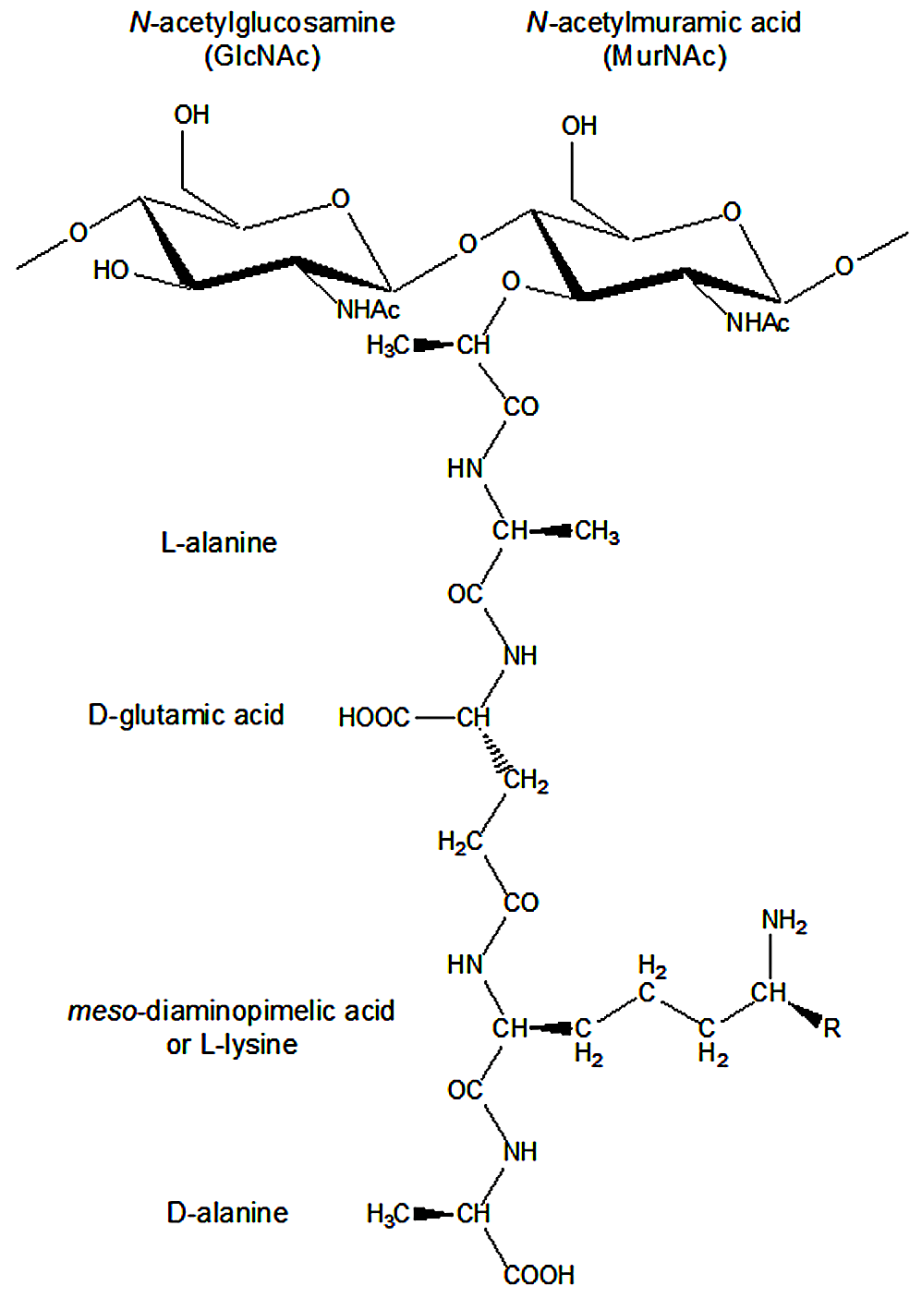
The monomer unit of the peptidoglycan structure. The disaccharide moiety is composed of the amino sugars *N*-acetylglucosamine (GlcNAc) and *N*-acetylmuramic (MurNAc) linked via a β-1,4 glycosidic bond. The amino acid at position 3 of the stem peptide is *meso*-diaminopimelic acid (R =  COOH) in most Gram-negative bacteria and l-lysine (R = H) in most Gram-positive bacteria.

The bacterial cell wall plays an integral role in withstanding stress from external and internal forces in addition to maintaining the shape of bacteria. As such, the cell wall is essential for cell viability due to its overarching function in providing physical support for the cytoplasmic membrane. The cell wall of bacteria is mainly composed of a cross-linked polymer known as peptidoglycan (PG). PG contains glycan chains and peptide stems, and its monomer unit consists of a disaccharide tetrapeptide (Fig. 1) [1]. Its synthesis is divided into three main steps. In the first step, the nucleotide sugar-linked precursors UDP-*N*-acetylmuramyl-pentapeptide (UDP-MurNAc-pentapeptide) and UDP-*N*-acetylglucosamine (UDP-GlcNAc) are synthesized in the cytoplasm. In the second step, precursor lipid intermediates (lipids I and II) are synthesized at the cytoplasmic membrane. The polymerization of newly synthesized disaccharide-peptide units and incorporation into the growing PG by penicillin-binding proteins (PBPs) is the third and final step of the pathway [2].

**Figure 2:**
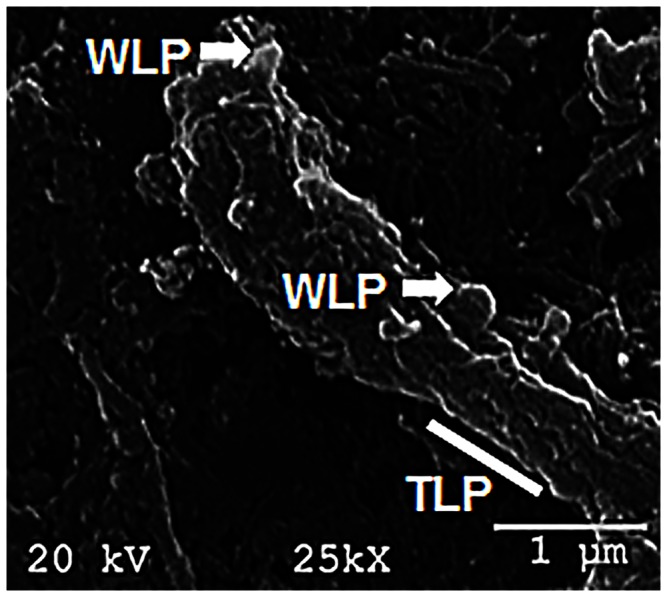
Scanning electron microscopy of *V. spinosum* DSM 4136^T^. The white arrows show the wart-like prosthecae (WLP) and the white bar depicts a tube-like prosthecae (TLP). The picture was taken at 25 K magnification. The scale bar is 1 µm.

*Verrucomicrobium spinosum* is a Gram-negative heterotrophic bacterium that is generally found in fresh water and soil. The morphology of *V. spinosum* is very interesting in that it possesses protruding wart-like and tube-like appendages known as prosthecae that are an extension of the cell membrane (Fig. 2). The bacterium has garnered a lot of interest from the scientific community due to its close evolutionarily relationship with bacteria from the genus *Chlamydia* [3]. Annotation of the genome suggests that the bacterium employs a protein secretion system known as Type III that is involved in pathogenicity [4]. A recent study shows that *V. spinosum* is pathogenic to *Drosophila melanogaster* and *Caenorhabditis elegans* [5].

*V. spinosum* was found to employ the recently discovered l,l-diaminopimelate aminotransferase (DapL) pathway [6], [7], [8], [9] as the sole route for the synthesis of diaminopimelate (A_2_pm) and l-lysine (l-Lys), based on biochemical and bioinformatical evidence [10]. In the anabolism of PG, the penultimate intermediate in the l-lysine biosynthesis pathway, *meso*-diaminopimelate (*meso*-A_2_pm), serves as one of the cross-linking amino acids in Gram-negative bacteria, and l-Lys serves the same purpose in many Gram-positive bacteria [11].

The enzyme UDP-*N*-acetylmuramoyl-l-alanyl-d-glutamate:*meso*-2,6-diaminopimelate ligase (MurE) (E.C. 6.3.2.15) catalyzes the addition of the third amino acid residue to the peptide stem of PG in the cytoplasmic step of PG synthesis. In most bacteria, this third residue is either *meso*-A_2_pm or l-Lys (Fig. 1). In particular species, other amino acids can be found, such as l-ornithine, *meso*-lanthionine, l,l-A_2_pm, l-diaminobutyric acid or l-homoserine [1], [12], [13]. Since the third residue in the bacterial cell wall is involved in PG cross-linking, the lack of or incorrect substrate incorporation into the PG macromolecule can lead to improperly constructed PG and ultimately to cell death via lysis due to inability of the bacterium to maintain osmotic pressure [14], [15].

Here we report the first characterization of a Mur ligase from the genus *Verrucomicrobium,* namely MurE from *V. spinosum* (MurE_Vs_). *In vivo* analysis demonstrates that the enzyme is able to functionally complement an *Escherichia coli* strain that harbors a mutation in the *murE* gene. Using *in vitro* analyses, we show that MurE_Vs_ is a *meso*-A_2_pm-adding enzyme. Furthermore, we present a structural analysis of the enzyme using protein sequence alignment and homology modeling, which shows that key amino acids for substrate binding and/or catalysis are conserved in MurE_Vs_. Together, these experiments contribute to the further understanding of the kinetic, physical and structural properties of the Mur ligase involved in the synthesis of PG from the organism *V. spinosum*. Finally, *V. spinosum* PG was purified and analyzed; its composition in which A_2_pm is one of the main constituents is similar to that of most Gram-negative bacteria.

## Materials and Methods

### *V. spinosum* growth conditions

*V. spinosum* DSM 4136^T^ was cultured in R2A medium at 26°C [10].

### PCR amplification and cloning of the *V. spinosum murE* open reading frame (ORF) for protein expression and purification

The open reading frame annotated by the locus tag (VspiD_010100019130) UDP-*N*-acetylmuramoyl-l-alanyl-d-glutamate:*meso*-2,6-diaminopimelate ligase was amplified by PCR. The following forward and reverse primers were used: *murE*_Vs_-forward 5′-CACC***ATG***ACCATTTTGCGCGATCTTATCGAGGGT-3′ and *murE*Vs-reverse 5′-GTCGAC ***TCA***CTGACGGTCATCCCTCCTTTGGCGTGC-3′ (the underlined sequence represents the restriction enzyme site used to facilitate sub-cloning of the ORF while the bold and italicized sequences represent initiation and termination codons). The PCR reaction contained 12 pmol of forward and reverse primers, 1 mM MgSO_4_, 0.5 mM of each of the four deoxynucleotide triphosphates, 0.5 ng of genomic DNA and 1 unit of Platinum *Pfx* DNA polymerase (Invitrogen Corporation, Carlsbad, CA, USA). PCR conditions were: 1 cycle at 94°C for 2 min, followed by 30 cycles of 94°C for 15 s, 60°C for 30 s and 72°C for 2 min. The *murE* PCR fragment was ligated into the plasmid pET100D/topo (Invitrogen Corporation, Carlsbad, CA, USA) to produce the plasmid pET100D::*murE*_Vs_. The recombinant protein encoded by this plasmid carries a MRGSHHHHHHGMASMTGGQQMGRDLYDDDDKDHPFT sequence containing a hexa-histidine tag derived from the pET100D plasmids at the amino terminus. To confirm the fidelity of the PCR reaction, the *murE* ORF was sequenced from pET100D using the T7 promoter primer, 5′-TAATACGACTCACTATAGGG-3′ and the T7 reverse primer, 5′-TAGTTATTGCTCAGCGGTGG-3′. The cloned *mur*E ORF was 100% identical to the sequences deposited in the Integrated Microbial Genomes public database (http://img.jgi.doe.gov/cgi-bin/w/main.cgi).

### Protein expression and purification of the recombinant MurE_vs_

The *E. coli* BL21-CodonPlus® (DE3)-RIPL (Agilent Technologies, USA) strain was transformed with the plasmid pET100D::*mur*E_Vs_ and grown in LB broth containing 50 µg⋅mL^−1^ ampicillin and 34 µg⋅mL^−1^ chloramphenicol at 37°C to an OD_600_ of 0.5. Protein expression was induced in 1 L of culture using isopropyl β-D-1-thiogalactopyranoside (IPTG) to a final concentration of 0.5 mM for 8 h at 20°C. The cell pellet was lysed by sonication in a buffer consisting of 50 mM sodium phosphate, pH 8.0, and 300 mM NaCl. The soluble extract was incubated with 1 mL bed volume of TALON Metal Affinity Resin (Clontech, Mountain View CA, USA) for 30 min at 4°C. The resin was washed 5 times with 30 mL of sonication buffer containing 10 mM imidazole for 15 min each. The enzyme was eluted with 10 mL of sonication buffer containing 250 mM imidazole. The hexa-histidine tag was not removed after protein purification. The pure protein was concentrated in an Amicon Ultra 10,000 molecular weight cutoff filter unit replacing the elution buffer with 20 mM potassium phosphate, pH 7.2, 1 mM dithiothreitol (DTT), 1 mM EDTA and 10% (v/v) glycerol. The protein concentration was determined by quantitative amino acid analysis as described below.

### Preparation of substrates and MurE activity assay

UDP-MurNAc-l-Ala-d-Glu, UDP-MurNAc-l-Ala-d-[^14^C]Glu and the three isomers of A_2_pm were prepared according to published procedures [16], [17], [18].

The standard MurE activity assay measured the formation of UDP-MurNAc-l-Ala-γ-d-[^14^C]Glu-*meso*-A_2_pm in mixtures (final volume, 40 µL) containing 100 mM Tris-HCl, pH 9.6, 30 mM MgCl_2_, 0.25 mg⋅mL^−1^ bovine serum albumin, 5 mM ATP, 150 µM UDP-MurNAc-l-Ala-d-[^14^C]Glu (350 Bq), 150 µM *meso*-A_2_pm, and enzyme (20 µL of an appropriate dilution in 20 mM phosphate buffer, pH 7.2, containing 1 mM DTT). After 30 min at 37°C, the reaction was stopped by the addition of glacial acetic acid (8 µL), followed by lyophilization. The radioactive substrate and product were separated on a Nucleosil 100 C_18_ 5 U column (150×4.6 mm; W. R. Grace S. A.) using 50 mM ammonium formate, pH 4.4, as the mobile phase at a flow rate of 0.6 mL⋅min^−1^. Radioactivity was detected with a flow detector (model LB506-C1, Berthold) using the Quicksafe Flow 2 scintillator (Zinsser Analytic) at 0.6 mL⋅min^−1^. Quantification was performed with the Radiostar software (Berthold).

Identical assay conditions were used when the l,l and d,d isomers of A_2_pm were tested as substrates. With lysine or ornithine, l-[^14^C]-Lys or l-[^14^C]-Orn, respectively, was used as the labeled substrate; in that case, radioactive substrate and product were separated by thin-layer chromatography on silica gel plates LK6D (Whatman) using 1-propanol/ammonium hydroxide/water (6∶3∶1; v/v) as the mobile phase, and the radioactive spots were located and quantified with a radioactivity scanner (Rita Star, Raytest Isotopenmeβgeräte GmbH).

### Determination of the kinetic constants

For the determination of the kinetic constants, the same assay was used with various concentrations of one substrate and fixed concentrations of the others. In all cases, the enzyme concentration was chosen so that substrate consumption was <20%, the linearity being ensured within this interval even at the lowest substrate concentration. Data were fitted to the equation *v* =  *V*_max_ *S*/(*K*_m_+*S*) by the Levenberg-Marquardt method [19], where *v* is the initial velocity and *S* is the substrate concentration, and values ± standard deviation at 95% of confidence were calculated. The MDFitt software developed by M. Desmadril (UMR 8619, CNRS, Orsay, France) was used for this purpose.

### Sequence alignment and homology modeling

A multiple amino acid sequence alignment between the Mur ligase enzymes of *V. spinosum* (ZP_02928794.1), *Mycobacterium tuberculosis* (CCE37632.1), *E. coli* (NP_414627.1) *Chlamydia trachomatis* (NP_219774.1) and *Pectobacterium carotovorum* (ZP_03831119.1) was generated using ClustalW2 (http://www.ebi.ac.uk/Tools/msa/clustalw2/) with the Gonnet scoring matrix.

The homology model of the MurE_Vs_ protein was generated using the SWISS-MODEL Protein Modeling Server [20], [21], [22] (http://swissmodel.expasy.org/) using the *E. coli* MurE structure as a template PDB id: 1E8C [23], which was identified using a PSI-BLAST search of the MurE_Vs_ protein sequence against proteins in the Protein Data Bank using the web server: (http://blast.ncbi.nlm.nih.gov/). The model was examined by hand for clashes and appropriate geometry using the visualization software PyMOL (The PyMOL Molecular Graphics System, Schrödinger, LLC).

### Purification and analysis of *V. spinosum* PG

PG was prepared and analyzed essentially according to Mengin-Lecreulx *et al.* [24]. Cells from 1 L of culture were harvested at 4°C and resuspended in 4% (w/v) sodium dodecyl sulfate (SDS) (10 mL⋅g^−1^ of cell wet weight) under constant and vigorous stirring at 100°C for 30 min. The suspension was incubated overnight at 25°C followed by centrifugation for 1 h at 17,000 rpm. The pellet containing crude PG was washed 5 times with 10 mL of sterile water and stored in water for further analysis.

Half of the preparation was used to obtain purified PG. Briefly, the following treatments at 37°C were performed: (i) overnight incubation with 0.05% (w/v) pancreatin in 0.1 M potassium phosphate buffer (pH 7.4); (ii) overnight incubation with 0.02% (w/v) pronase in 0.01 M Tris-HCl buffer (pH 7.4); (iii) overnight incubation with 0.02% (w/v) trypsin in 0.02 M potassium phosphate buffer (pH 7.4). Finally, after centrifugation and several washes with 8 M lithium chloride containing 0.1 M EDTA, and water, the pellet was stored in water. Aliquots of crude and purified PGs were hydrolyzed as described below.

### Amino acid and hexosamine analysis

Samples were hydrolyzed in 6 M HCl containing 0.05% (v/v) 2-mercaptoethanol at 105°C for 24 h (proteins), or in 6 M HCl at 95°C for 16 h (PG). After evaporation of the acid, the pellet was dissolved with 67 mM trisodium citrate-HCl buffer (pH 2.2) and injected into a Hitachi L-8800 amino acid analyzer equipped with a 2620MSC-PS column (ScienceTec). Amino acids and hexosamines were detected after post-column reaction with ninhydrin.

## Results

### The genome of *V. spinosum* contains the full complement of genes necessary for the *de novo* synthesis of peptidoglycan

The *V. spinosum* genome was searched from the Integrated Microbial Genomes (IMG) database (http://www.jgi.doe.gov/) using the annotated PG synthesis pathway from Kyoto Encyclopedia of Genes and Genomes (KEGG). The search resulted in the identification of 20 genes that are known to be involved in PG metabolism. Importantly, the search identified orthologs of all the genes necessary for the *de novo* synthesis of PG in *V. spinosum* (Table 1).

**Table 1:** List of genes involved in PG metabolism of *V. spinosum* DSM 4136^T^.

Locus Tag	Protein Symbol	Annotated Gene Product Name	EC Number
VspiD_010100024635	PBP	d-Alanyl-d-alanine carboxypeptidase-class C	3.4.16.4
VspiD_010100022270	PBP	Multimodular transpeptidase-transglycosylase-class A	2.4.1.129
VspiD_010100006740	PBP	Penicillin-binding protein 1C- class A	2.4.1.129
VspiD_010100020475	PBP	Penicillin-binding protein 2-class A	2.4.1.129
VspiD_010100007940	PBP	Peptidoglycan transpeptidase-class B	2.4.1.129
VspiD_010100017450	PBP	Peptidoglycan transpeptidase-class B	2.4.1.129
VspiD_010100019135	PBP	Peptidoglycan synthetase FtsI-class B	2.4.1.129
VspiD_010100018680	PBP	Cell elongation specific d,d-transpeptidase- class B	2.4.1.129
VspiD_010100019120	MraY	Phospho-*N*-acetylmuramoyl-pentapeptide-transferase	2.7.8.13
VspiD_010100011745	MurA	UDP-*N*-acetylglucosamine 1-carboxyvinyltransferase	2.5.1.7
VspiD_010100019100	MurG	UDP-*N*-acetylglucosamine-*N*-acetylmuramoyl-(pentapeptide) pyrophosphoryl-undecaprenol *N*-acetylglucosamine transferase	2.4.1.227
VspiD_010100019125	MurF	UDP-*N*-acetylmuramoyl-tripeptide:d-alanyl-d-alanine ligase	6.3.2.10
VspiD_010100018175	Ddl	d-Alanine:d-alanine ligase	6.3.2.4
VspiD_010100019115	MurD	UDP-*N*-acetylmuramoyl-l-alanine:d-glutamate ligase	6.3.2.9
VspiD_010100019130	MurE	UDP-*N*-acetylmuramoyl-l-alanyl-d-glutamate:*meso*-2,6-diaminopimelate ligase	6.3.2.13
VspiD_010100026230	UppP	Undecaprenyl pyrophosphate phosphatase	3.6.1.27
VspiD_010100018130	MurB	UDP-*N*-acetylenolpyruvoylglucosamine reductase	1.1.1.158
VspiD_010100018130	MurC	UDP-*N*-acetylmuramate:l-alanine ligase	6.3.2.8
VspiD_010100000100	AlaR	Alanine racemase	5.1.1.1
VspiD_010100008415	MurI	Glutamate racemase	5.1.1.3

The annotated gene product names are from NCBI (www.ncbi.nlm.nih.gov/protein/) queried of February 28, 2013. The pencillin-binding proteins (PBP) class designations are denoted by activity based on **p**rotein **fam**ily (pfam) domains. Class A and class B PBPs are high-molecular mass PBPs while class C PBPs are low-molecular mass PBPs. Class A PBPs are predicted to have both transglycosylase and transpeptidase activities; class B PBPs are predicted to have only transpeptidase activity; class C PBPs are predicted to have d,d-carboxypeptidase activity.

### Identification of the MurE ortholog from *V. spinosum*

The orthologous MurE protein from *V. spinosum* was initially identified using the MurE protein sequence from *C. trachomatis* (NP_219774) as a query. The BlastP algorithm from the Integrated Microbial Genomes (IMG) database was employed. The search resulted in the identification of a putative MurE from *V. spinosum* annotated by the locus tag VspiD_010100019130, which is 37% identical to the *C. trachomatis* MurE [10].

### Overproduction and purification of murE Ligase from *V. spinosum*

**Figure 3:**
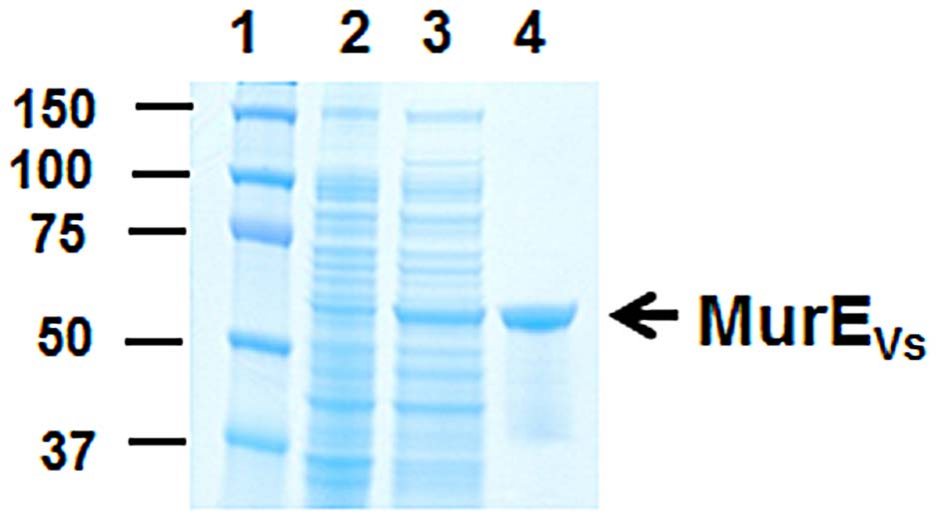
Expression and purification of recombinant MurE_Vs_ using His-tag affinity chromatography. Lane (1) protein makers (kDa); Lane (2) 10 µg of soluble protein from uninduced cells; Lane (3) 10 µg of soluble protein from induced cells; Lane (4) 1 µg of purified recombinant MurE_Vs_. The proteins were resolved on 10% (w/v) acrylamide gel and were stained using Coomassie blue.

The *murE*_Vs_ gene was cloned into the pET100D/topo plasmid, allowing expression of the protein with an N-terminal short peptide extension comprising a hexa-histidine tag (see Materials and Methods). *E. coli* BL21 DE3-CodonPlus-RIPL cells, transformed with the resulting vector pET100D::*murE*_Vs_, were grown and subjected to IPTG induction. Extraction and purification afforded 2.5 mg of MurE_Vs_ per liter of culture. The protein was homogenous by SDS-PAGE: a band at ∼59 kDa could be observed, in keeping with a calculated molecular mass of 59,578 Da. (Fig. 3). Its identity was further confirmed by MALDI-TOF mass spectrometry: peaks at *m/z* 59,568 and 29,774 Da, corresponding to the [MH]^+^ and [M+2H]^2+^ ions, respectively, were observed (Fig. S1).

### Kinetic properties of the MurE ligase from *V. spinosum*

The optimal pH, temperature and magnesium concentration for MurE_Vs_ were found to be 9.6, 44–46°C, and 30 mM, respectively. *In vitro* assays were thus performed at pH 9.6 and with 30 mM MgCl_2_, the usual temperature of 37°C being used. With *meso*-A_2_pm and UDP-MurNAc-l-Ala-d-Glu as substrates, the maximum velocity was 36±2 µmol⋅min^−1^⋅mg^−1^. The *K*_m_ values for the substrates were: ATP, 290±70 µM; UDP-MurNAc-l-Ala-d-Glu, 24±6 µM; and *meso*-A_2_pm, 17±3 µM. The enzyme proved to be stereospecific for *meso*-A_2_pm, since the rate of incorporation of the l,l or d,d isomer was <1% that of the *meso* isomer. No incorporation of l-lysine or l-ornithine could be detected, even with a significant amount of purified recombinant enzyme (Table 2).

**Table 2:** Specificity of MurE_Vs_ for the amino acid substrate.

Substrate	Enzymatic activity (μmol.min^−1^.mg^−1^)^a^
*meso*-A_2_pm	36
l,l-A_2_pm	0.18
d,d-A_2_pm	0.043
l-Lysine	ND^b^
l-Ornithine	ND

a Determined as described in Materials and Methods with fixed concentrations of ATP (5 mM), UDP-MurNAc-l-Ala-d-Glu (0.15 mM) and amino acid (0.15 mM).

b ND, no activity detected after 30 minutes with 11 µg of enzyme.

### Sequence alignment and homology modeling

To identify conserved regions of the enzyme and motifs employed during catalysis, a multiple amino acid sequence alignment was performed between MurE enzymes from *V. spinosum, M. tuberculosis, E. coli, C. trachomatis* and *P. carotovorum* using ClustalW2 (http://www.ebi.ac.uk/Tools/msa/clustalw2/). Ten of the 16 putative active site residues thought to be involved in substrate binding were conserved among all five sequences. The key DNPR motif [23], [25], [26] which comprises residues 409–412 in the *V. spinosum* sequence, was identical across all five sequences.

To examine both the sequence and consider the consequences of differences within the MurE_Vs_ active site, we developed a MurE_Vs_ homology model. Using the MurE_Vs_ amino acid sequence as a template, we performed a PSI-BLAST search against proteins with known structure in the Protein Data Bank. The top hits were the MurE enzymes from *M. tuberculosis* and *E. coli,* with an identity of 38% and 37%, respectively. We chose the *E. coli* MurE structure (PDB id: 1E8C) as a template since this ortholog was well characterized [23], and generated a homology model for MurE_Vs_ using SWISS-MODEL (http://swissmodel.expasy.org/). The QMEAN score of the homology model was 0.55 (range is between 0 to 1) and the QMEAN Z score is −3.51 (Fig. S2) [27].

**Figure 4:**
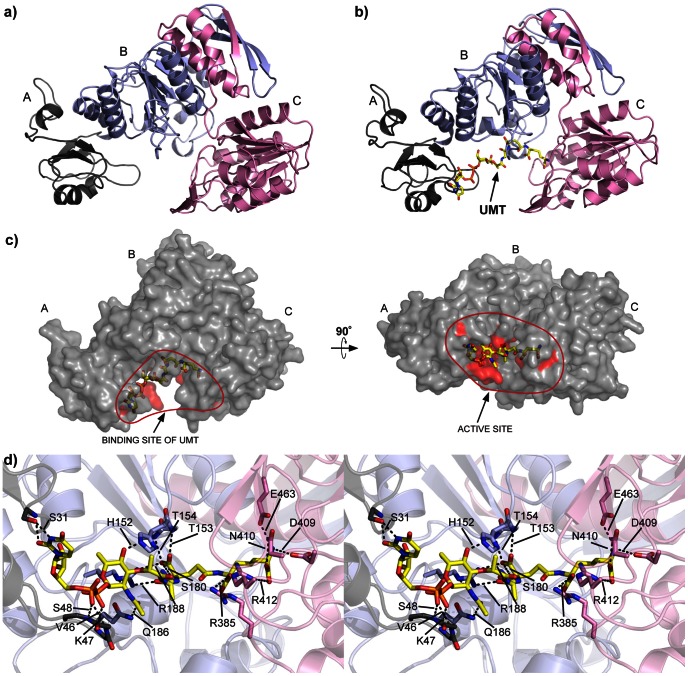
Homolgy model of MurE_Vs_. (a) The homology model of MurE_Vs_ highlighting domains A (grey), B (violet) and C (pink). (b) Shows the structure model of MurE_Vs_ bound to UDP-MurNAc-tripeptide (UMT) product (yellow). (c) Active site residue hypothesized to bind to UMT product is shown in red. The structure has been rotated 90° on the right panel for the better viewing of the binding pocket. (d) Cross eye stereo view showing the interaction between amino acid residues of the binding site and UMT product.

As annotated in the MurE_Ec_ structure, the MurE_Vs_ homology model is predicted to have three domains: A, B and C (Fig. 4a). Domain A comprises residues 1–84 and consists of a four-stranded parallel β-sheet, compared to the five-stranded β-sheet in the template MurE_Ec_, and the β-sheet is flanked by two helices, as in template structure. Domain B comprises residues 85–289 and consists of a central six-stranded parallel β-sheet surrounded by six α-helices. The MurE_Ec_ template has seven α-helices and in the homology model the seventh α-helix is broken into two small helices. Additionally, there are two antiparallel strands that interact with domain C, which comprises residues 290–507 and consists of a six-stranded β-sheet with five parallel strands and one anti-parallel strand flanked by six α-helices.

**Figure 5:**
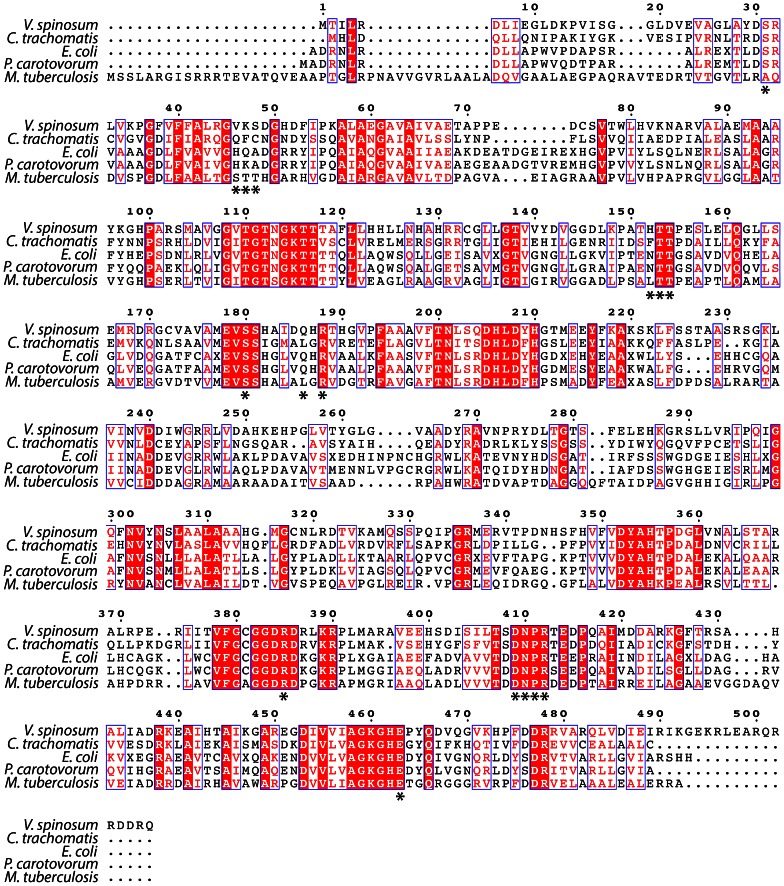
Multiple amino acid sequence alignment of five representative sequences of MurE. The residues that are predicted to be involved in binding in the active site are marked with a star below the sequence. The sequence identity score against MurE from *V. spinosum* was: *C. trachomatis,* 37%; *E. coli,* 35%; *P. carotovorum*, 36%; and *M. tuberculosis*. The multiple amino acid sequence alignment figure was generated using the ESPript 2.2 server (http://espript.ibcp.fr/ESPript/cgi-bin/ESPript.cgi).

An overlay of our MurE_Vs_ homology model with the ligand-bound MurE_Ec_ template structure highlights how the UDP-MurNAc-tripeptide (UMT) product (albeit in the conformation that fits within the MurE_Ec_ active site) is proposed to interact with residues in the putative active site of MurE_Vs_ (Fig. 4b and c). A comparison of the active site binding residues suggests that all three domains of MurE_Vs_ are involved in the interaction with the product (Fig. 4d). Most interactions already found between MurE_Ec_ and UMT [23] are conserved with MurE_Vs_. In particular, hydrogen bonds between the ε-carboxyl group of *meso*-A_2_pm and N410 and R412 are predicted. These two H-bonds have been proposed to be responsible for the *meso*-A_2_pm/l-Lys discrimination [23]. A direct sequence comparison between the MurE_Vs_ and MurE_Ec_ active sites suggests that only 12 out of 16 active site residues are conserved in MurE_Vs_ (Fig. 5). However, three of the five non-conserved residues (K47, S48, H152, *V. spinosum* numbering) employ their main-chain atoms for binding and may therefore be more tolerant to mutation with minimal effects on substrate/product binding.

### Isolation and analysis of *V. spinosum* PG

Peptidoglycan has been indirectly detected in *V. spinosum* in a recent study using *in situ* probing *via* florescent D-amino acids [28]. To directly ascertain that *V. spinosum* does in fact possess authentic PG; cells were submitted to boiling SDS, a treatment used to isolate PG from other bacteria [24], [29]. Analysis of the SDS-insoluble material (Table 3) revealed that it contained Mur and A_2_pm; Mur is a specific component of all PGs, and A_2_pm is found in PG from Gram-negative bacteria. However, this crude PG was contaminated with proteogenic amino acids. Most of these amino acids could be removed by protease treatment. Molar ratios of the main PG constituents in the purified polymer were: Glu, 0.9; Ala, 1.5; A_2_pm, 1.1; Mur, 0.8; GlcN, 1.0; other amino acids had molar ratios ≤0.06 (Table 3). Therefore, this experiment shows that *V. spinosum* possesses a PG that is similar to the one of *E. coli* [24] and indeed other Gram-negative bacteria [1]. From the quantitative determination of A_2_pm in the crude PG preparation, an A_2_pm content of the PG of *V. spinosum* of 1.5×10^−11^ µmol/cell was estimated. This is of the same order of magnitude as the one found for *E. coli* PG (8.2×10^−12^ µmol/cell [24].

**Table 3:** Analysis of crude and purified PG from *V. spinosum* DSM 4136^T^.

Constituent	Molar ratio (Calculated with GlcN = 1.0)	
	Crude PG^a^	Purified PG^a^
Asp	1.22	0.04
Thr	0.62	0.03
Ser	0.63	0.03
Mur	0.79	0.80
Glu	2.33	0.91
Pro	0.47	0.02
Gly	0.95	0.06
Ala	2.47	1.51
Val	0.54	0.04
A_2_pm	1.02	1.10
Met	0	0
Ile	0.42	0.03
Leu	0.86	0.05
Tyr	0.34	0.03
Phe	0.39	0.03
GlcN	1.0	1.0
Lys	0.76	0.04
His	0.22	0.02
Arg	0.43	0.02

a Crude and purified PG designate the macromolecule before and after, respectively, treatment with pancreatin, pronase and trypsin (see Materials and Methods).

## Discussion

The heterotrophic Gram-negative bacterium *V. spinosum* has recently garnered significant interest from the scientific community, since the genome has been sequenced, annotated and is publically available. In addition, the bacterium was found to be pathogenic towards *D. melanogaster* and *C. elegans*, two model invertebrate organisms [5].

A recent study from our laboratories confirmed the presence of the plant-like biosynthetic pathway for diaminopimelate and l-lysine in *V. spinosum* through the partial characterization of the enzyme l,l-diaminopimelate aminotransferase (DapL) [10]. In the same study, we identified the MurE ortholog and showed that the enzyme was able to functionally complement an *E. coli* mutant that harbors a mutation in the *murE* gene [10].

The genus *Verrucomicrobium* is evolutionarily related to the genus *Chlamydia* [3]. Interestingly, we were able to identify all the genes that are involved in the *de novo* anabolism of PG from the annotated genome of *V. spinosum* (Table 1). The MurE ortholog from *Chlamydia trachomatis* was identified and was shown to be an authentic MurE enzyme, even though PG cannot be detected from the bacterium using methods developed thus far [26]. Unlike *C. trachomatis*, we were able to isolate and detect PG from *V. spinosum* in addition to quantifying all the major components of the macromolecule. *V. spinosum* is an attractive candidate model organism to address questions relating to: i) the chlamydial PG paradox; and ii) the feasibility and plausibility of whether the newly discovered DapL enzyme is a potential target for antibiotic development given the fact the enzyme is involved in the synthesis of both PG and lysine.

MurE_Vs_ shares 37% and 35% amino acid identity to the MurE orthologs from *C. trachomatis* and *E. coli*, respectively. With regards to the substrate specificity of the enzyme, MurE_Vs_ resembles that of the *C. trachomatis* and *E. coli* orthologs by showing preference for *meso*-A_2_pm. The enzyme incorporated very weakly the two other stereoisomers of A_2_pm; it was unable to incorporate l-lysine and l-ornithine, two structurally related diamine compounds. Therefore, MurE_Vs_ is highly specific for *meso*-A_2_pm.

The enzyme's optimum catalytic profile with respect to pH, temperature and [Mg^2+^] was examined to define optimum assay conditions and also gauge its similarity with other known MurE enzymes. MurE_Vs_ displays maximum activity at pH 9.6, which is slightly higher than those found in *E. coli* (pH 8.0–9.2) and *C. trachomatis* (pH 8.0–8.6) Mur ligases [15]. The optimal temperature for MurE_Vs_ (44–46°C) seems somewhat high but difficult to compare with other orthologs and paralogs since this parameter is almost never mentioned. These unusual values for MurE_Vs_ might be attributed to environmental factors such as the natural habitat(s) of the organism. As for the optimal [Mg^2+^] concentration, it falls within the range (5–100 mM) found for *E. coli* and *C. trachomatis* Mur ligases [15], [26], [30].

The maximum velocity of 36 µmol⋅min^−1^⋅mg^−1^ for the MurE_Vs_ using saturating levels of all substrates is approximately 110, 26 and 14 times more than those of MurE_Ct_, MurE_Ec_ and MurE from *Pseudomonas aeruginosa*, respectively [15], [26], [31]. Whereas the higher specific activity of MurE_Vs_ with respect to MurE_Ct_ can easily be explained by the fact that *Chlamydiae* are slow-growing, primarily intracellular organisms [26], we have no explanation for the difference between MurE_Vs_ and the orthologs from *E. coli* and *P. aeruginosa*.

Primary sequence analysis showed that MurE_Vs_ contains ten out of the sixteen amino acids that make up the active site of the enzyme including the DNPR motif. The DNPR motif is conserved among MurEs that have been experimentally authenticated. A homology model, based on the well characterized MurE_Ec_ enzyme (PDB id: 1E8C), was developed to examine the sequence further and consider the consequences of differences within the MurE_Vs_ active site. The MurE_Vs_ enzyme is likely to comprise three domains, A, B and C, each of which contribute amino acid residues to the active site. Nearly all of the active site moieties (10 of 16) known to interact with the substrates and products are conserved in the MurE_Vs_ active site. Overall, the homology model is entirely consistent with our validated function of MurE_Vs_ and suggests that the enzyme binds the substrates in a similar way to other known MurE enzymes.

Even though the diaminopimelate/l-lysine pathway have been the subject and focus of numerous studies regarding the development of antibiotics, no novel antibiotics have been developed or identified thus far that target any enzyme belonging to the four variants of the anabolic pathways [6], [32]. To this end, we are interested in the essentiality of the DapL enzyme in eubacteria that defines one of the four anabolic variants identified thus far. The enzyme converts tetrahydrodipicolinate to l,l-diaminopimelate in one step circumventing three enzymatic steps in the *E. coli* acyl pathways [6]. l,l-Diaminopimelate is subsequently converted to the *meso* isomer by an epimerase; this facilitates the synthesis of lysine *via* a decarboxylation reaction for protein synthesis in addition to cell wall biosynthesis *via* MurE in many Gram-positive bacteria. The inhibition of DapL or other enzymes in the diaminopimelate/l-lysine pathway would affect bacterial growth in two different ways. First the bacteria will be unable to grow because of the lack of protein synthesis due to the absence of l-lysine. Second, PG biosynthesis will be impaired due to the lack of *meso*-A_2_pm. Presumably, this will result in a bacteriostatic effect, as already observed for other enzymes of the diaminopimelate/l-lysine pathway [33], [34].

The genomes of animals and particularly humans do not possess the genetic machinery to facilitate the biosynthesis of diaminopimelate/l-lysine *de novo*. Therefore, animals must acquire l-lysine through dietary means. Thus there is a unique opportunity to assess the essentiality of enzymes that are important for cell wall and protein synthesis from eubacteria. *V. spinosum* is an attractive model bacterial system based on the fact that the organism is closely related to *Chlamydia*, which was found to use the DapL pathway to diaminopimelate/l-lysine. Bioinformatic analysis shows that the sequenced and annotated genomes of bacteria belonging to the genus *Chlamydia* contain putative *dapL* orthologs (data not shown). *V. spinosum* is aerobic and facile to culture using commercially available media because it is not an obligate intracellular bacterium as is the case with *Chlamydia*. Importantly, the bacterium is not pathogenic to mammals based on what we currently know. Since the genome of the organism can be genetically modified using transposon mutagenesis, analysis of genes that are essential for *V. spinosum* that are involved in the diaminopimelate/l-lysine biosynthesis can be the focus of future studies [10], [35].

Here we present the identification and characterization of the first Mur ligase namely, MurE from the bacterium *V. spinosum*. Bioinformatic and biochemical analyses provide evidence that the bacterium is able to synthesize PG *de novo*. *In vivo* analysis shows that MurE_Vs_ is an authentic *meso*-A_2_pm adding enzyme. This was further validated by *in vitro* analyses that show that the kinetic and physical properties are consistent with MurE orthologs that have been experimentally confirmed. Finally, primary amino acid sequence and structural analysis based on protein modeling show that key amino acids that are involved in substrate binding and or catalysis are conserved in MurE_Vs_.

## Supporting Information

Figure S1 **MALDI-TOF mass spectrometry analysis of purified MurE_Vs_. Matrix: sinapinic acid.** Peaks with *m/z* ratios consistent with the His_6_-tagged protein (calculated mass, 59,578 Da) are shown.
(TIF)

Figure S2 **Homology model quality statistics.** The cartoon structure shows the quality of model by coloring the residues according to the error. The coloring is from blue (reliable region) to red (potentially unreliable region). The residue error plot depicts the local model reliability with estimated pre-residue inaccuracies along the sequence.
(TIF)

## References

[1] Vollmer W, Blanot D, de Pedro MA (2008) Peptidoglycan structure and architecture. FEMS Microbiol Rev 32: 149–167. 18194336 10.1111/j.1574-6976.2007.00094.x

[2] Scheffers DJ, Pinho MG (2005) Bacterial cell wall synthesis: new insights from localization studies. Microbiol Mol Biol Rev 69: 585–607. 16339737 10.1128/MMBR.69.4.585-607.2005 PMC1306805

[3] Wagner M, Horn M (2006) The Planctomycetes, Verrucomicrobia, Chlamydiae and sister phyla comprise a superphylum with biotechnological and medical relevance. Curr. Opin. Biotechnol 17: 241–249. 16704931 10.1016/j.copbio.2006.05.005

[4] Hueck CJ (1998) Type III protein secretion systems in bacterial pathogens of animals and plants. Microbiol Mol Biol Rev 62: 379–433. 9618447 10.1128/mmbr.62.2.379-433.1998 PMC98920

[5] Sait M, Kamneva OK, Fay DS, Kirienko NV, Polek J, Shiasus-Hiza MM, et al (2011) Genomic and experimental evidence suggests that *Verrucomicrobium spinosum* interacts with eukaryotes. Front. Microbiol 2: 211 doi:10.3389/fmicb.2011.00211 22022322 10.3389/fmicb.2011.00211 PMC3196152

[6] Hudson AO, Singh BK, Leustek T, Gilvarg C (2006) An L,L-diaminopimelate aminotransferase defines a novel variant of the lysine biosynthesis pathway in plants. Plant Physiol 140: 292–301. 16361515 10.1104/pp.105.072629 PMC1326051

[7] McCoy AJ, Adams NE, Hudson AO, Gilvarg C, Leustek T, et al (2006) Diaminopimelate aminotransferase a trans-kingdom enzyme shared by Chlamydia and plants for synthesis of diaminopimelate/lysine. Proc. Natl. Acad. Sci. USA 103: 17909–17914. 17093042 10.1073/pnas.0608643103 PMC1693846

[8] Hudson AO, Gilvarg C, Leustek T (2008) Biochemical and phylogenetic characterization of a novel diaminopimelate biosynthesis pathway in prokaryotes identifies a diverge from of L,L-diaminopimelate aminotransferase. J. Bacteriol 190: 3256–3263. 18310350 10.1128/JB.01381-07 PMC2347407

[9] Liu Y, White RH, Whitman WB (2010) Methanococci use the diaminopimelate aminotransferase (DapL) pathway for lysine biosynthesis. J. Bacteriol 192: 3304–3310. 20418392 10.1128/JB.00172-10 PMC2897669

[10] Nachar VR, Savka FC, McGroty SE, Donovan KA, North RA, et al (2012) Genomic and biochemical analysis of the diaminopimelate and lysine biosynthesis pathway in *Verrucomicrobium spinosum*: identification and partial characterization of L,L-diaminopimelate aminotransferase and UDP-N-acetylmuramoylalanyl-D-glutamyl-2,6-meso-diaminopimelate ligase. Front. Microbiol 3: 183. 22783236 10.3389/fmicb.2012.00183 PMC3390587

[11] Hutton CA, Perugini MA, Gerrard JA (2007) Inhibition of lysine biosynthesis: an evolving antibiotic strategy. Mol. Biosyst 3: 458–465. 17579770 10.1039/b705624a

[12] Schleifer KH, Kandler O (1972) Peptidoglycan types of bacterial cell wall and their taxonomic implications. Bacterol Rev 36: 407–477. 10.1128/br.36.4.407-477.1972 PMC408328 4568761

[13] Barreteau H, Kovač A, Boniface A, Sova M, Gobec S, et al (2008) Cytoplasmic steps of peptidoglycan biosynthesis. FEMS Microbiol Rev 32: 168–207. 18266853 10.1111/j.1574-6976.2008.00104.x

[14] Mengin-Lecreulx D, Falla T, Blanot D, van Heijenoort J, Adams DJ, et al (1999) Expression of the *Staphylococcus aureus* UDP-*N*-acetylmuramoyl- L-alanyl-D-glutamate:L-lysine ligase in *Escherichia coli* and effects on peptidoglycan biosynthesis and cell growth. J. Bacteriol 181: 5909–5914. 10498701 10.1128/jb.181.19.5909-5914.1999 PMC103616

[15] Patin D, Boniface A, Kovač A, Hervé M, Dementin S, et al (2010) Purification and biochemical characterization of Mur ligases from *Staphylococcus aureus* . Biochimie 92: 1793–1800. 20659527 10.1016/j.biochi.2010.07.009

[16] Babič A, Patin D, Boniface A, Hervé M, Mengin-Lecreulx D, et al.. (2007) Chemoenzymatic synthesis of the nucleotide substrates of the Mur ligases, 1–4. In D. Kikelj, 5th Joint Meeting on Medicinal Chemistry, 17 to 21 June, Portoro?, Slovenia. Medimond Srl, Bologna, Italy.

[17] Michaud C, Mengin-Lecreulx D, van Heijenoort J, Blanot D (1990) Over-production, purification and properties of the uridine-diphosphate-*N*-acetylmuramoyl-l-alanyl-d-glutamate:*meso*-2,6-diaminopimelate ligase from *Escherichia coli* . Eur. J. Biochem 194: 853–861. 2269304 10.1111/j.1432-1033.1990.tb19479.x

[18] van Heijenoort J, Bricas E (1968) Contribution à l′étude des isomères de l′acide α,α′-diaminopimélique. Bull. Soc. Chim. Fr 7: 2828–2831.

[19] Press WH, Flannery BP, Teukolsky SA, Vetterling WT (1986) Numerical recipes:.The art of scientific computing, Cambridge University Press, Cambridge, UK.

[20] Arnold K, Bordoli L, Kopp J, Schwede T (2006) The SWISS-MODEL workspace: a web-based environment for protein structure homology modelling. Bioinformatics 22: 195–201. 16301204 10.1093/bioinformatics/bti770

[21] Kiefer F, Arnold K, Künzli M, Bordoli L, Schwede T (2009) The SWISS-MODEL repository and associated resources. Nucleic Acids Research 37: 387–392. 10.1093/nar/gkn750 PMC2686475 18931379

[22] Peitsch MC (1995) Protein Modeling by E-mail. Nat Biotech 13: 658–660.

[23] Gordon E, Flouret B, Chantalat L, van Heijenoort J, Mengin-Lecreulx D, et al (2001) Crystal structure of UDP-N-acetylmuramoyl-L-alanyl-D-glutamate: *meso*-diaminopimelate ligase from *Escherichia coli* . J. Biol Chem 276: 10999–11006. 11124264 10.1074/jbc.M009835200

[24] Mengin-Lecreulx D, Flouret B, van Heijenoort J (1982) Cytoplasmic steps of peptidoglycan synthesis in *Escherichia coli* . J. Bacteriol 151: 1109–1117. 6125497 10.1128/jb.151.3.1109-1117.1982 PMC220385

[25] Boniface A, Bouhss A, Mengin-Lecreulx D, Blanot D (2006) The MurE synthetase from *Thermotoga maritima* is endowed with an unusual D-lysine adding activity. J Biol Chem 281: 15680–15686. 16595662 10.1074/jbc.M506311200

[26] Patin D, Bostock J, Blanot D, Mengin-Lecreulx D, Chopra I (2009) Functional and biochemical analysis of the *Chlamydia trachomatis* ligase MurE. J. Bacteriol 191: 7430–7435. 19820100 10.1128/JB.01029-09 PMC2786587

[27] Benkert P, Biasini M, Schwede T (2011) Toward the estimation of the absolute quality of individual protein structure models. Bioinformatics 27: 343–350. 21134891 10.1093/bioinformatics/btq662 PMC3031035

[28] Kuru E, Hughes HV, Brown PJ, Hall E, Tekkam S (2012) In situ probing of newly synthesized peptidoglycan in live bacteria with fluorescent d-amino acids. Angew. Chem Int. Ed. 51 : doi: 10.1002/anie.201206749 10.1002/anie.201206749 PMC3589519 23055266

[29] Girardin SE, Boneca IV, Viala J, Chamaillard M, Labigne A (2003) Nod2 is a general sensor of peptidoglycan through muramyl dipeptide (MDP) detection. J. Biol. Chem 278: 8869–8872. 12527755 10.1074/jbc.C200651200

[30] Patin D, Bostock J, Chopra I, Mengin-Lecreulx D, Blanot D (2012) Biochemical characterization of the chlamydial MurF ligase, and possible sequence of the chlamydial peptidoglycan pentapeptide stem. Arch. Microbiol 194: 505–512. 22231476 10.1007/s00203-011-0784-8

[31] Paradis-Bleau C, Lloyd A, Sanschagrin F, Maaroufi H, Clarke T (2009) *Pseudomonas aeruginosa* MurE amide ligase: enzyme kinetics and peptide inhibitor. Biochem. J. 421: 263–272. 19400768 10.1042/BJ20081395

[32] Cox RJ (1996) The DAP pathway to lysine as a target for antimicrobial agents. Nat. Prod. Rep 13: 29–43. 8919551 10.1039/np9961300029

[33] Berges DA, DeWolf DE, Dunn GL, Grappel SF, Newman DJ (1986) Peptides of 2-aminopimelic acid: antibacterial agents that inhibit diaminopimelic acid biosynthesis. J. Med. Chem 29: 89–95. 3079832 10.1021/jm00151a015

[34] Le Roux P, Blanot D, Mengin-Lecreulx D, van Heijenoort J (1991) Peptides containing 2-aminopimelic acid. Synthesis and study of *in vitro* effect on bacterial cells. Int. J. Peptide Protein Res 37: 103–111. 2019472

[35] Domman DB, Steven BT, Ward NL (2011) Random transposon mutagenesis of *Verrucomicrobium spinosum* DSM 4136(T). Arch. Microbiol 193: 307–312. 21184215 10.1007/s00203-010-0666-5

