# Supplementary figures and images for: Biochemical Characterization of UDP-N-acetylmuramoyl-L-alanyl-D-glutamate: meso-2,6-diaminopimelate ligase (MurE) from Verrucomicrobium spinosum DSM 4136T
Source: PLoS One. 2013 Jun 13;8(6):e66458. doi: 10.1371/journal.pone.0066458 (PMC3681970; doi:10.1371/journal.pone.0066458)

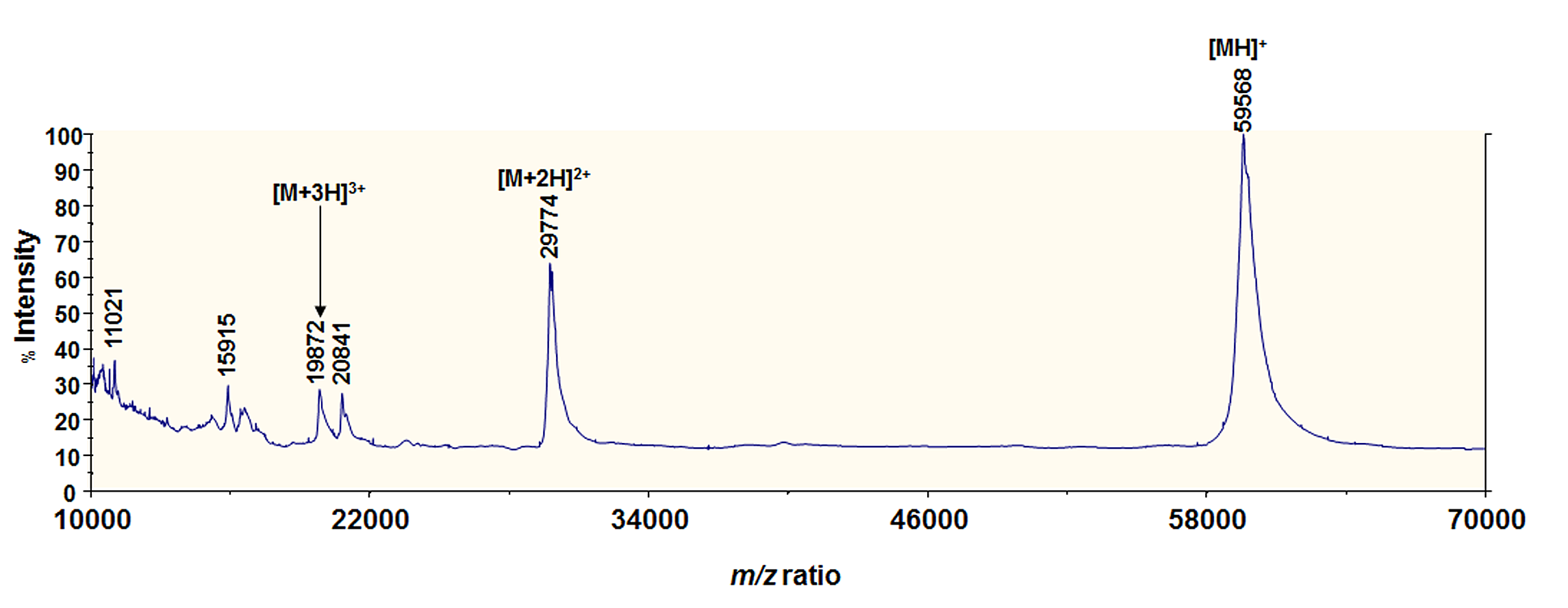

Supplement: Figure S1 — MALDI-TOF mass spectrometry analysis of purified MurEVs. Matrix: sinapinic acid. Peaks with m/z ratios consistent with the His6-tagged protein (calculated mass, 59,578 Da) are shown. (TIF) [file pone.0066458.s001.tif]

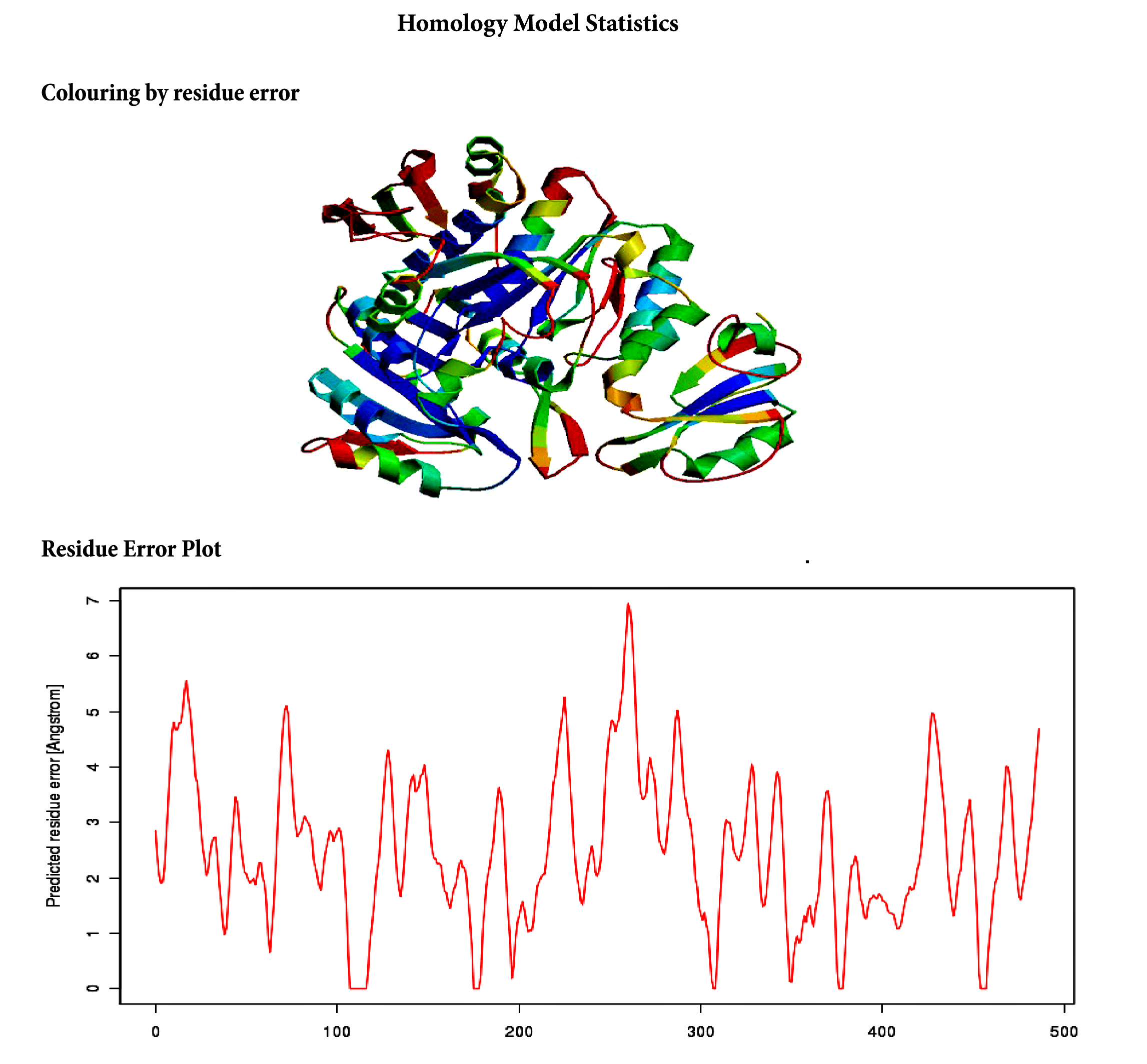

Supplement: Figure S2 — Homology model quality statistics. The cartoon structure shows the quality of model by coloring the residues according to the error. The coloring is from blue (reliable region) to red (potentially unreliable region). The residue error plot depicts the local model reliability with estimated pre-residue inaccuracies along the sequence. (TIF) [file pone.0066458.s002.tif]
